# The non-high-density lipoprotein cholesterol to high-density lipoprotein cholesterol ratio as a predictive indicator of CKD risk in NAFLD patients: NHANES 2017–2020

**DOI:** 10.3389/fnut.2024.1501494

**Published:** 2024-12-24

**Authors:** Yong-Qiang Fan, Hao Wang, Pei-Pei Wang, Zhi-Yong Shi, Yan Wang, Jun Xu

**Affiliations:** ^1^Liver Transplantation Center, The First Hospital of Shanxi Medical University, Taiyuan, China; ^2^Department of Respiratory, The Second Hospital of Shanxi Medical University, Taiyuan, China

**Keywords:** NAFLD, fibrosis, CKD, NHHR, lipid metabolism, NHANES

## Abstract

**Background:**

Non-alcoholic fatty liver disease (NAFLD) and chronic kidney disease (CKD) are both closely related to dyslipidemia. However, the relationship between dyslipidemia in patients with NAFLD and CKD is not yet clear. The non-high-density lipoprotein cholesterol to high-density lipoprotein cholesterol ratio (NHHR) is an innovative and comprehensive lipid index. The purpose of this study was to investigate the correlation between NHHR and CKD risk in NAFLD patients with or without fibrosis.

**Methods:**

This study used data from the National Health and Nutrition Examination Survey (NHANES) from 2017 to 2020 for analysis, including a total of 4,041 subjects diagnosed with NAFLD. Among the NAFLD subjects, 3,315 individuals without liver fibrosis and 726 individuals with fibrosis. Weighted multivariate linear regression, weighted logistic regression, restricted cubic spline (RCS) curves, and subgroup analysis were used to evaluate the correlation between NHHR and CKD in patients with NAFLD.

**Results:**

Our findings indicate that in NAFLD subjects without liver fibrosis, the highest tertile of NHHR, as compared to the lowest tertile, was inversely related to glomerular filtration rate (eGFR) (*β*: −2.14, 95% CI: −3.97, −0.32, *p* < 0.05) and positively related to CKD (OR: 1.67, 95% CI: 1.12, 2.49, *p* < 0.05). No significant associations were observed between NHHR and eGFR, urinary albumin to creatinine ratio (ACR) in NAFLD subjects with liver fibrosis. The RCS revealed a linear relationship between NHHR and ACR, CKD in NAFLD subjects without liver fibrosis, while a U-shaped relationship was observed between NHHR and ACR, CKD in NAFLD subjects with liver fibrosis.

**Conclusion:**

In patients with non-fibrotic NAFLD, a significantly elevated NHHR is closely associated with an increased risk of CKD and shows a linear relationship with CKD. In patients with fibrotic NAFLD, NHHR shows a U-shaped relationship with CKD. LD, Our findings underscore the practical utility of NHHR as a biomarker for early risk stratification of CKD in patients with NAFLD.

## 1 Introduction

Non-alcoholic fatty liver disease (NAFLD) is the most prevalent chronic liver disease, affecting approximately 25% of the global adult population, according to statistics (1, 2). The spectrum of NAFLD encompasses nonalcoholic fatty liver, nonalcoholic steatohepatitis, progressing to liver fibrosis, cirrhosis, and ultimately hepatocellular carcinoma (3). NAFLD is closely linked to insulin resistance, chronic inflammation, and metabolic disorder (4). In this context, NAFLD, as a multisystem disease, not only impairs the normal function of the liver but also affects the kidneys, cardiovascular system, pancreas, and other organs (4, 5).

Chronic kidney disease (CKD) denotes abnormalities in kidney structure or function, with its diagnosis relying on the detection of markers for kidney damage and the duration of such damage (6). Research indicates that patients with NAFLD have a CKD incidence rate ranging from 20 to 55%, significantly higher than the 5 to 35% observed in non-NAFLD patients (7). Moreover, the incidence of CKD differs among patients with NAFLD at varying stages, the progress of NAFLD was positively associated with incidence of CKD (8). The aforementioned studies suggest that NAFLD is a significant contributor to the development of CKD. However, the initial symptoms of patients with NAFLD are often subtle, and some patients are already in the fibrosis stage when they seek medical care. By then, their risk of developing CKD will increase significantly. Therefore, early and timely identification of high-risk populations among NAFLD patients is a critical step in preventing the occurrence of CKD in NAFLD patients.

Numerous researches have identified the intricate link between dyslipidemia and CKD. Dyslipidemia has been identified as a potentially driving factors of CKD (9, 10). The dyslipidemia of CKD patients primarily consists of elevated levels of triglycerides and triglyceride-rich lipoprotein particles, along with reduced levels of high-density lipoprotein cholesterol (HDL-C) (9). In the cardiovascular system, HDL can exert a protective effect through the reverse cholesterol transport (6). However, the protective effect of HDL on CKD is still controversial. In certain research, excessively high levels of HDL can also damage kidney function (11–13). Additionally, a prospective cohort study found that multiple lipids or lipoproteins, including triglycerides, high-density lipoprotein, and low-density lipoprotein, cannot be used as independent predictors of CKD (14). Based on the above research results, a single lipid or lipoprotein is not suitable as a biomarker for determining the severity and progression of CKD.

Non-HDL-C primarily comprises LDL-C, very low-density lipoprotein (VLDL), intermediate -density lipoprotein, and the cholesterol within lipoprotein (a) (15). The Non-HDL-C to HDL-C ratio (NHHR) serves as a new comprehensive index that includes multiple lipid particles related to atherosclerosis (16). Research has shown that compared to traditional lipid markers, NHHR exhibits higher diagnostic performance in predicting insulin resistance and metabolic syndrome (17). However, it is still unclear whether NHHR can be used to predict the risk of developing CKD in NAFLD patients. Therefore, utilizing data from the National Health and Nutrition Examination Survey (NHANES), this study aimed to uncover the relationship between the NHHR and the risk of developing CKD in NAFLD patients. This study hypothesized that there would be a strong association between the NHHR and the risk of developing CKD in NAFLD patients.

## 2 Methods

### 2.1 Study design

This study employed clinical data collected from NHANES database (2017–2020). Participants were interviewed in their homes, followed by physical examinations and laboratory tests at the Mobile Examination Center (MEC). NHANES was conducted with the approval of the Institutional Review Board of the National Center for Health Statistics in the United States and secured informed written consent from all participants (18).

### 2.2 Participants

**Figure 1:**
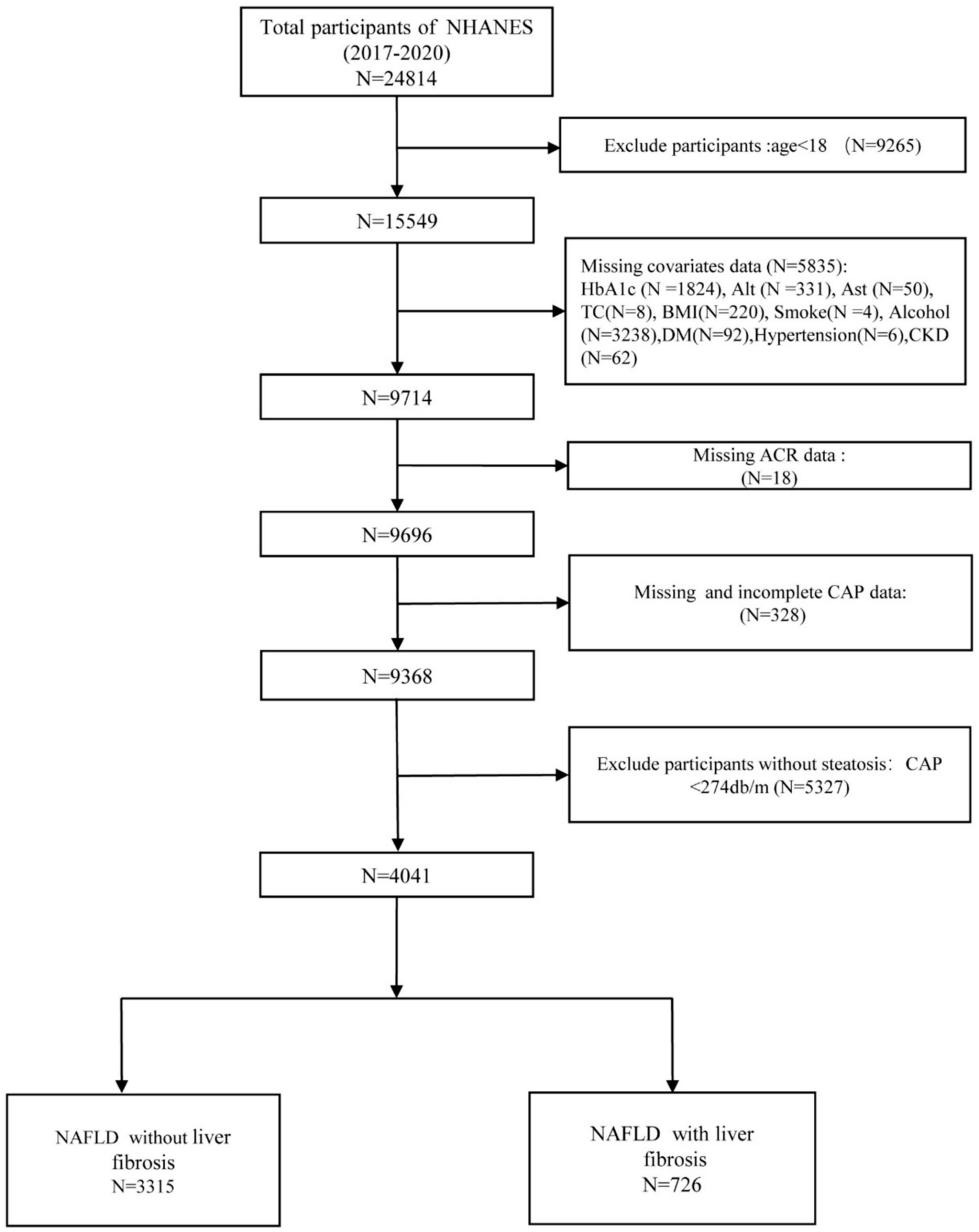
Flowchart of participant enrollment and exclusion in this study.

To evaluate the correlation between NHHR and CKD in NAFLD subjects with or without liver fibrosis, this study included subjects diagnosed with NAFLD and liver fibrosis. Consequently, a total of 24,814 participants were examined across four interview periods spanning from 2017 to 2020. The following participants were excluded from this study: (1) age < 18 years (*N* = 9,265); (2) missing covariate data (*N* = 5,835); (3) missing ACR data (*N* = 18); (4) missing CAP data (*N* = 328); and (5) participants without steatosis (*N* = 5,327). Ultimately, 4,041 participants were included in this study. In the NAFLD population, there were 3,315 individuals without liver fibrosis and 726 individuals with fibrosis (Figure 1).

This study evaluated liver steatosis and fibrosis using controllable attenuation parameters (CAP) and liver stiffness measurement (LSM) determined by vibration control transient elastography. CAP ≥ 274 dB/m is defined as NAFLD (19). LSM above 8kpa is defined as liver fibrosis, and below 8kpa is defined as no fibrosis (20).

### 2.3 Exposure variables

The exposure variable is NHHR, derived from the ratio of Non-HDL-C (mmol/L) to HDL-C (mmol/L). Non-HDL-C is calculated as the difference between total cholesterol (TC) and HDL-c in the blood (21). Subjects were categorized into three groups based on the third quartiles of NHHR: Q1 group (0.28, 2.49), Q2 group (2.49, 3.54), and Q3 group (3.54, 24.5). Outcome variables included eGFR, ACR, and CKD. In this study, CKD was defined as meeting any of the following criteria, as per guidelines: (1) glomerular filtration rate (eGFR) < 60 mL/min/1.73 m2, as calculated using the Chronic Kidney Disease Epidemiology Collaboration equation and (2) albuminuria ≥ 30 mg/g (22, 23).

### 2.4 Covariates

The covariates included in this study include age (years), sex (male or female), eth (Mexican American, other Hispanic, Non-Hispanic White, Non-Hispanic Black, and other races), BMI (kg/m^2^), smoke (former, never or now), alcohol (heavy, moderate, mild or never), Alt (mg/dL), Ast (mg/dL), HbA1c (%), TC (mmol/L), HDL-C (mmol/L), Non-HDL-C (mmol/L), ACR, DM (DM, IFG or no), Hypertension (yes or no), CKD (yes or no).

### 2.5 Statistical analysis

Considering the complex sampling design of the NHANES database, this study employed weighted approaches throughout the data analysis to ensure the generation of representative estimates reflective of the US national population. Continuous variables were expressed as means and standard errors, utilizing weighted *t*-tests. Categorical variables are expressed using *N* and weighted percentages (%), with differences compared using weighted chi-square tests.

Multiple linear regression and logistic regression were utilized to analyze the association between the third quartile of NHHR and eGFR, ACR, CKD, respectively. This study utilized unadjusted, minimally adjusted, and fully adjusted models for evaluation. Crude model: Single-factor linear and logistic regression models; Model 1: Adjusted for age and sex; Model 2: Further adjusted for eth, BMI, smoking, alcohol, TC, Alt, Ast, HbA1c, and hypertension.

RCS curve model was used to analyze the nonlinear relationship between NHHR and eGFR, ACR, CKD. Subsequently, subgroup analyses were performed to assess the stability of the association between NHHR and CKD across various stratifications, with the results visualized as forest plots. Subgroups were stratified by sex, eth, BMI, smoking, alcohol, DM, and hypertension. If the P for interaction across different stratifications is >0.05, it suggests the results are reliable across different subgroups; otherwise, it may indicate the presence of special populations (24, 25).

## 3 Results

### 3.1 Basic characteristics of participants

Table 1 presents the demographic characteristics of 4,041 NAFLD participants, with an average age of 49.26 years, 56.99% being male, and 43.01% being female. The majority of the subjects were Non-Hispanic White (62.84%). Compared to participants in the lowest tertile of NHHR, those in the higher tertile were typically younger, predominantly male, former or current smokers, heavy drinkers, with lower levels of HDL-C, higher levels of BMI, TC, Non-HDL-C, HbA1c, ALT, AST, ACR, and an increased prevalence of CKD (*p* < 0.05).

**Table 1:** Clinical characteristics based on the third quartile of NHHR.

Variable	Total	Q1	Q2	Q3	*p*
Age	49.26 (0.62)	52.47 (1.03)	49.18 (0.78)	46.30 (0.63)	<0.0001
Sex					<0.0001
Female	1798 (43.01)	743 (54.74)	636 (46.05)	419 (28.90)	
Male	2,243 (56.99)	605 (45.26)	709 (53.95)	929 (71.10)	
Eth					0.003
Mexican American	737 (12.58)	209 (10.51)	252 (12.55)	276 (14.56)	
Non-Hispanic Black	786 (8.31)	350 (11.26)	250 (7.72)	186 (6.11)	
Non-Hispanic White	1,446 (62.84)	480 (64.31)	495 (63.47)	471 (60.81)	
Other Hispanic	410 (6.67)	119 (5.87)	135 (6.39)	156 (7.71)	
Other Race	662 (9.60)	190 (8.05)	213 (9.87)	259 (10.81)	
BMI (kg/m^2^)	33.65 (0.24)	32.46 (0.35)	33.87 (0.32)	34.56 (0.26)	<0.0001
Smoke					<0.001
Former	1,014 (27.38)	363 (28.48)	321 (22.49)	330 (31.18)	
Never	2,360 (57.69)	822 (60.64)	792 (61.44)	746 (51.18)	
Now	667 (14.93)	163 (10.88)	232 (16.07)	272 (17.64)	
Alcohol					<0.001
Heavy	955 (25.34)	298 (23.08)	291 (24.18)	366 (28.65)	
Moderate	747 (19.29)	300 (21.71)	239 (20.04)	208 (16.25)	
Mild	1854 (46.46)	615 (49.33)	613 (43.28)	626 (46.89)	
Never	485 (8.91)	135 (5.88)	202 (12.50)	148 (8.22)	
TC (mmol/L)	4.98 (0.04)	4.38 (0.05)	4.91 (0.04)	5.62 (0.05)	<0.0001
HDL-C (mmol/L)	1.26 (0.01)	1.55 (0.02)	1.23 (0.01)	1.01 (0.01)	<0.0001
Non-HDL-C (mmol/L)	3.73 (0.04)	2.83 (0.04)	3.68 (0.03)	4.61 (0.04)	<0.0001
HbA1c (%)	5.90 (0.03)	5.85 (0.03)	5.82 (0.05)	6.02 (0.04)	0.003
Alt (mg/dL)	28.21 (0.46)	25.30 (0.63)	25.76 (0.50)	33.40 (0.95)	<0.0001
Ast (mg/dL)	23.54 (0.34)	23.45 (0.58)	21.89 (0.33)	25.24 (0.63)	<0.0001
ACR	33.27 (3.60)	23.15 (3.17)	20.12 (2.02)	55.85 (10.54)	0.003
eGFR	94.68 (0.78)	92.62 (1.49)	95.10 (0.98)	96.21 (0.83)	0.06
Hypertension					0.15
No	2047 (52.95)	664 (55.82)	674 (52.23)	709 (50.95)	
Yes	1994 (47.05)	684 (44.18)	671 (47.77)	639 (49.05)	
DM					0.15
DM	1,118 (23.72)	400 (24.77)	344 (22.01)	374 (24.43)	
IFG	404 (10.96)	136 (11.60)	135 (8.99)	133 (12.31)	
No	2,519 (65.31)	812 (63.63)	866 (69.00)	841 (63.26)	
CKD					0.04
No	3,301 (84.07)	1,102 (86.84)	1,100 (84.38)	1,099 (81.14)	
Yes	740 (15.93)	246 (13.16)	245 (15.62)	249 (18.86)	

All the variables are presented as the mean (SE) or *n* (%). BMI, body mass index; HbA1c, glycated haemoglobin; TC, total cholesterol; HDL-C, high-density lipoprotein cholesterol; Alt, alanine aminotransferase; Ast, aspartate aminotransferase; eGFR, estimated glomerular filtration rate; ACR, urinary albumin to creatinine ratio.

### 3.2 Association between NHHR and CKD in NAFLD without liver fibrosis

We observed that NHHR was significantly associated with eGFR and CKD risk in individuals with NAFLD without fibrosis (Table 2). When NHHR is considered as a continuous variable, it shows significant correlation with both eGFR and CKD. In the unadjusted model (Crudel Model), NHHR was positively correlated with eGFR (*β* = 0.95, 95%CI: 0.10, 1.80, *p* < 0.05). In Model 1, NHHR was negatively correlated with eGFR (*β* = −0.56, 95%CI: −0.96, −0.16, *p* < 0.05). It is noteworthy that a significant negative correlation still exists in the fully adjusted model (Model 2) (*β* = −0.93, 95%CI: −1.26, −0.60, *p* < 0.0001). In addition, NHHR was positively correlated with increased risk of CKD, and this positive correlation was statistically significant in both Model 1 (*β* = 1.16, 95%CI: 1.04, 1.30) and Model 2 (*β* = 1.11, 95%CI: 1.01, 1.22). However, in the fully adjusted model, we did not observe a significant correlation between NHHR and ACR (*p* > 0.05). Similar results were also shown when NHHR was analyzed as a categorical variable (tertile). Compared with the lowest tertile of NHHR, a negative correlation was still observed between the highest tertile of NHHR and eGFR (*β* = −2.14, 95%CI: −3.97, −0.32, *p* < 0.05). In the adjusted multivariate model, NHHR was positively correlated with CKD (Model 1: 1.16, 95% CI: 1.04, 1.30, *p* < 0.05; Model 2: OR: 1.11, 95% CI: 1.01, 1.22, *p* < 0.05).

**Table 2:** The correlation between NHHR and CKD in NAFLD without fibrosis.

	Crude model		Model 1		Model 2	
	OR/*β* (95%CI)	*p*	OR/*β* (95%CI)	*p*	OR/*β* (95%CI)	*p*
eGFR						
NHHR (continuous)	0.95 (0.10, 1.80)	0.03	−0.56 (−0.96, −0.16)	0.01	−0.93 (−1.26, −0.60)	<0.0001
NHHR (quartile)						
Q1	Ref		Ref		Ref	
Q2	2.83 (−0.70, 6.36)	0.11	−0.06 (−2.22, 2.10)	0.96	0.04 (−1.97, 2.06)	0.96
Q3	4.34 (1.07, 7.61)	0.01	−1.11 (−2.95, 0.73)	0.23	−2.14 (−3.97, −0.32)	0.02
P for trend		0.01		0.23		0.02
ACR						
NHHR (continuous)	7.37 (0.00, 14.74)	0.05	8.18 (1.01, 15.34)	0.03	4.48 (−0.79, 9.75)	0.09
NHHR (quartile)						
Q1	Ref		Ref		Ref	
Q2	−2.86 (−11.71, 5.99)	0.52	−1.29 (−9.83, 7.26)	0.76	−2.53 (−11.88, 6.82)	0.58
Q3	29.84 (−0.33, 60.01)	0.05	33.38 (4.41, 62.35)	0.03	24.36 (2.35, 51.07)	0.07
P for trend		0.05		0.02		0.07
CKD						
NHHR (continuous)	1.07 (0.97, 1.18)	0.18	1.16 (1.04, 1.30)	0.01	1.11 (1.01, 1.22)	0.03
NHHR (quartile)						
Q1	Ref		Ref		Ref	
Q2	1.22 (0.81, 1.82)	0.34	1.46 (0.94, 2.25)	0.09	1.33 (0.78, 2.28)	0.28
Q3	1.42 (0.98, 2.05)	0.06	2.06 (1.40, 3.04)	<0.001	1.67 (1.12, 2.49)	0.02
P for trend		0.06		<0.001		0.01

Data are expressed as *β* or OR and 95% confidence interval (CI). Crude model: unadjusted model; Model 1: adjusted for age and sex. Model 2: adjusted for age, sex, eth, BMI, smoking, alcohol, Alt, Ast, HbA1c, hypertension, and DM.

### 3.3 The correlation between NHHR and CKD NAFLD with liver fibrosis

As shown in Table 3, when NHHR was considered as a continuous variable, NHHR was significantly positively correlated with CKD risk in both the unadjusted model and Model 1 [Crude Model: *β*(95CI%) 1.18 (1.02, 1.38); Model 1: *β*(95CI%) 1.24 (1.08, 1.41), *p* < 0.05]. However, in the fully adjusted model, this significance disappeared (*p* > 0.05). We did not find a significant relationship between NHHR and eGFR, ACR. When NHHR is treated as a categorical variable, the results are consistent with those previously observed.

**Table 3:** Correlation between NHHR and CKD in NAFLD with fibrosis.

	Crude model		Model 1		Model 2	
	95%CI	*p*	95%CI	*p*	95%CI	*p*
eGFR						
NHHR (continuous)	0.95 (−0.32, 2.22)	0.14	0.51 (−0.20, 1.23)	0.15	0.57 (−0.04, 1.18)	0.07
NHHR (quartile)						
Q1	Ref		Ref		Ref	
Q2	−2.06 (−7.55, 3.42)	0.45	−3.24 (−8.06, 1.59)	0.18	−1.57 (−5.88, 2.75)	0.46
Q3	1.94 (−4.26, 8.14)	0.53	−1.09 (−4.95, 2.77)	0.57	0.72 (−2.92, 4.36)	0.68
p for trend		0.44		0.72		0.59
ACR						
NHHR (continuous)	47.2 (−4.91, 99.32)	0.07	49.38 (−3.72, 102.49)	0.07	48.51 (−4.82, 101.84)	0.07
NHHR (quartile)						
Q1	Ref		Ref		Ref	
Q2	−10.35 (−32.67, 11.98)	0.35	−10.74 (−32.98, 11.50)	0.33	21.32 (−53.92, 11.28)	0.19
Q3	38.37 (−22.94, 99.68)	0.21	39.44 (−22.02, 100.90)	0.20	34.29 (−29.56, 98.15)	0.28
P for trend		0.18		0.18		0.24
CKD						
NHHR (continuous)	1.18 (1.02, 1.38)	0.03	1.24 (1.08, 1.41)	0.003	1.14 (0.98, 1.31)	0.09
NHHR (quartile)						
Q1	Ref		Ref		Ref	
Q2	0.96 (0.47, 1.98)	0.91	1.04 (0.45, 2.39)	0.92	0.85 (0.40, 1.78)	0.65
Q3	1.30 (0.71, 2.39)	0.39	1.56 (0.83, 2.92)	0.16	1.05 (0.53, 2.12)	0.88
P for trend		0.37		0.14		0.81

Data are expressed as *β* or OR and 95% confidence interval (CI). Crude model: unadjusted model; Model 1: adjusted for age and sex. Model 2: adjusted for age, sex, eth, BMI, smoking, alcohol, Alt, Ast, HbA1c, hypertension, and DM.

### 3.4 Nonlinear relationships

**Figure 2:**
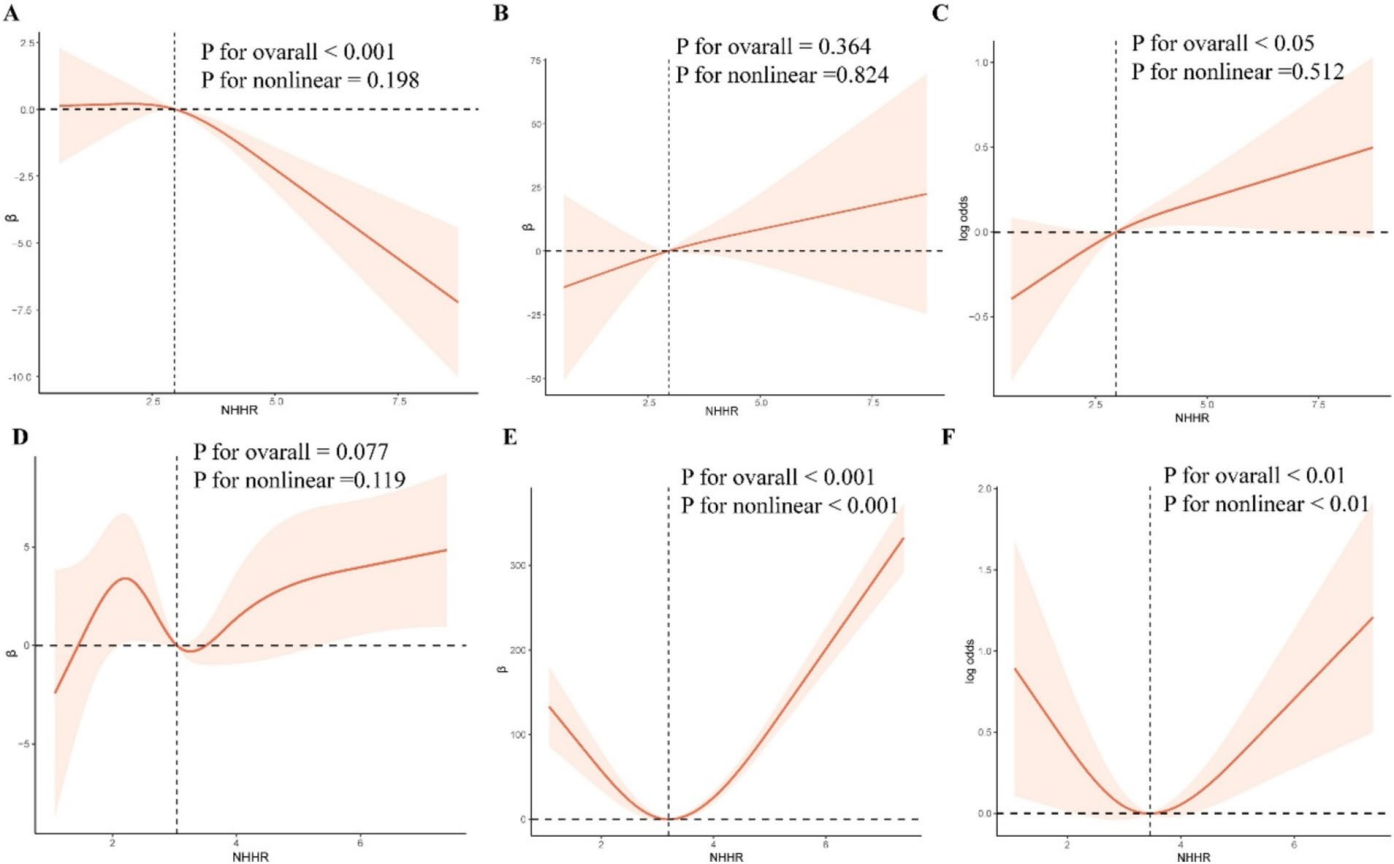
RCS curve model. After adjusting for confounding variables, RCS was used to analyze the nonlinear relationship between NHHR and eGFR, ACR, and CKD.

The RCS curve model was used to further explore the possible nonlinear relationship between NHHR and eGFR, ACR, and CKD (Figure 2). After adjusting all confounding variables in Model 2, the results showed that there was a linear relationship between NHHR and ACR, CKD in NAFLD without liver fibrosis (P overall >0.05, P nonlinear >0.05). A U-shaped nonlinear relationship between NHHR and ACR, CKD was observed in NAFLD with liver fibrosis (thresholds were 3.20 and 3.45, respectively).

### 3.5 Subgroup analysis

**Figure 3:**
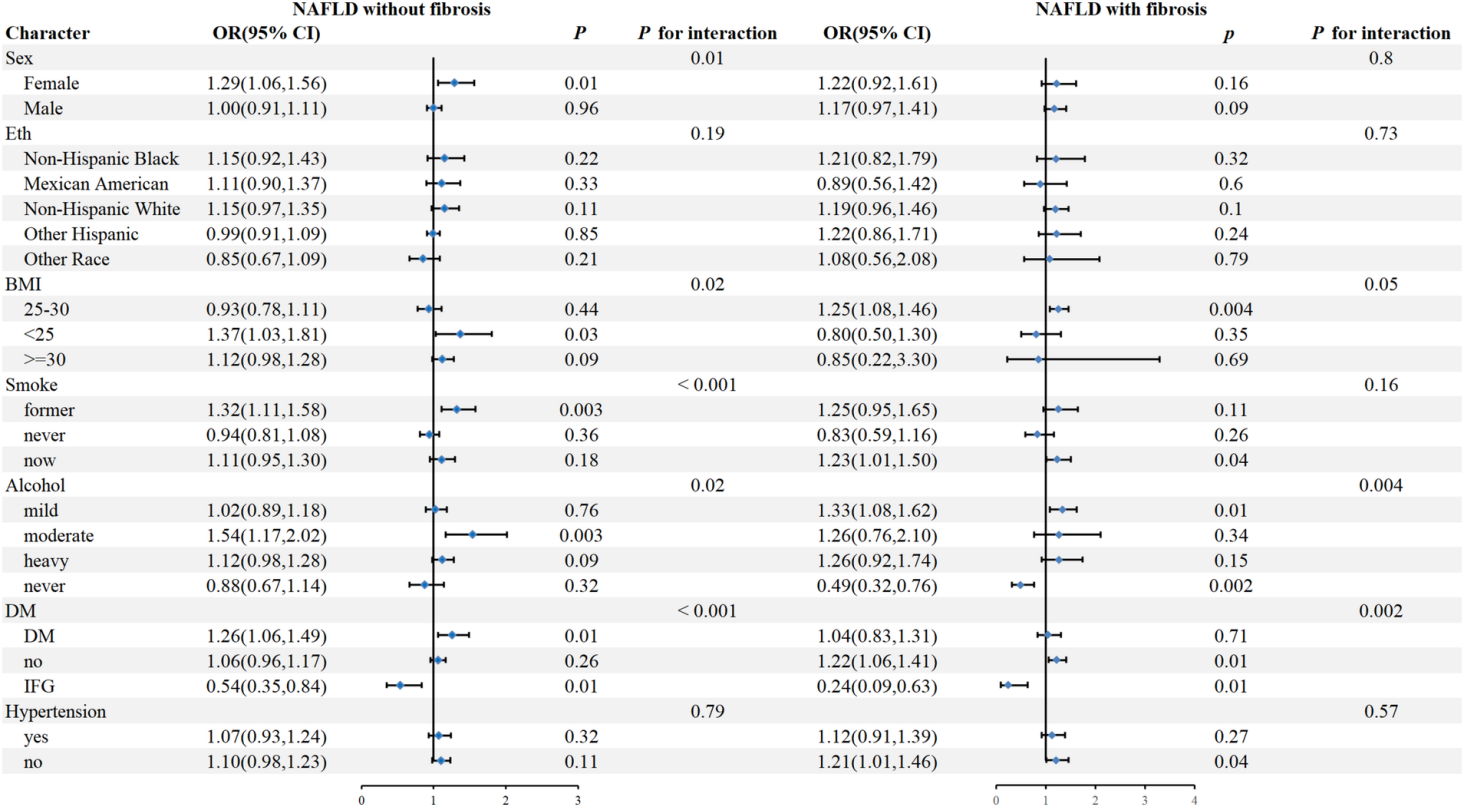
Subgroup analysis.

To further assess the impact of NHHR on outcome measures, we analyzed NHHR as a continuous variable in subgroups defined by sex, eth, BMI, smoke, alcohol, DM, and hypertension (Figure 3). The results showed that in NAFLD individuals without fibrosis, a positive correlation between NHHR and CKD was observed in female, BMI <25, previous smokers, moderate alcohol, DM, and IFG patients (P for interaction <0.05), while there was no significant interaction in the subgroups of eth and hypertension. In addition, in NAFLD individuals with fibrosis, NHHR was significantly associated with CKD in those who were mildly or never alcohol, non-DM, and IFG (P for interaction <0.05), while no significant interaction was observed in any other subgroups.

## 4 Discussion

Our study from this large cross-sectional study reveals the association between NHHR and the risk of developing CKD in patients with NAFLD. Our study indicates a U-shaped relationship between NHHR and CKD in NAFLD patients with liver fibrosis as well as a linear relationship with CKD in NAFLD patients without liver fibrosis. Our findings underscore the practical utility of NHHR as a biomarker for early risk stratification of CKD in patients with NAFLD.

Due to the global prevalence of obesity, type 2 diabetes, and hypertension, the incidence rate of NAFLD and CKD has rapidly increased in recent decades. Four similarities imply a substantial link between NAFLD and CKD: both are common in chronic disease populations, both are closely related to metabolic disorders, both are linked with an increased risk of cardiovascular events, and there are gender differences in incidence rates (4, 26, 27). Although, the overlap in pathogenesis and risk factors between NAFLD and CKD makes it difficult to distinguish the causal relationship between the two diseases. However, many studies still clearly indicate that the presence of NAFLD increases the likelihood of CKD, and the increase in the incidence rate of CKD is directly proportional to the severity of NAFLD (8, 28–30). Therefore, NAFLD is an independent risk factor for the development of CKD.

Patients with NAFLD frequently exhibit dysregulated lipid metabolism, with their lipid profiles typically displaying elevated levels of non-HDL cholesterol and reduced levels of HDL cholesterol. Insulin resistance reduces the sensitivity of organs like the liver and adipose tissue to insulin, resulting in heightened fatty acid synthesis within the liver (31, 32). To maintain the lipid metabolism homeostasis, the liver increases the synthesis and secretion of VLDL triglyceride (VLDL-TG) (33–35). After VLDL-TG enters the blood circulation, the triglyceride in VLDL are hydrolyzed under the action of lipoprotein lipase (36). At the same time, VLDL receives cholesterol esters (CE) from HDL. As the exchange continues, the TG content in VLDL decreases, while the CE content increases. VLDL eventually becomes IDL and LDL, which have higher density and smaller diameter (37). Additionally, multiple studies found that HDL lever in NAFLD patients are often lower than normal (38, 39). The exchange of triglycerides and cholesterol esters between HDL and non-HDL is regulated by cholesteryl ester transfer protein (CETP). In patients with NAFLD, the increased activity of CETP promotes the production and degradation of TG-rich HDL, resulting in a decrease in HDL-C levels (37, 40). The increase in Non-HDL-C levels, along with the decrease in HDL-C levels, jointly contribute to the elevation of NHHR indicators in patients with NAFLD. The increase of NHHR can be used as a characteristic marker of dyslipidemia and insulin resistance in patients with NAFLD.

In terms of the risk of CKD, multiple studies have indicated that the presence of insulin resistance greatly increases the risk of CKD in patients with NAFLD (5, 41, 42). As mentioned above, an increase in NHHR can be considered a sign of insulin resistance (17, 43). Therefore, insulin resistance may be one of the key mechanisms explaining NHHR as an assessment of the risk of developing CKD in NAFLD patients. In addition, HDL is well-known for its antioxidant, anti-inflammatory, and maintaining endothelial function properties. The deficiency of HDL promotes the infiltration of inflammatory cells and the dysfunction of endothelial cells, which contributes to the progression of kidney diseases (44). Meanwhile, In patients with non-alcoholic fatty liver disease (NAFLD), the imbalance between increased secretion and clearance of VLDL-TG leads to hypertriglyceridemia, which in turn leads to an increase in the number of small dense LDL (sd-LDL) particles (45, 46). The sd-LDL particles are easily oxidized by free radicals, and the oxidized low density lipoprotein (ox-LDL) has strong lipotoxicity (47). Ox-LDL can induce the onset of CKD by enhancing the activity of the fibrotic signaling pathway, fostering the infiltration of inflammatory cells, and encouraging epithelial-mesenchymal transition in renal tubular epithelial cells (48–51). Increased Non-HDL-C levels and decreased HDL-C levels are two important risk factors for CKD. In summary, the dysregulation of lipid metabolism caused by NAFLD is involved in the development of CKD. Compared with other markers, NHHR, as a comprehensive lipid metabolism marker, integrates key lipid information related to dyslipidemia and can better reflect the overall lipid status of patients. Our research results further found that NHHR can be used to predict the risk of developing CKD in patients with NAFLD.

Within this research, the stratification groups of the subjects were divided into three tertiles based on the NHHR. Our study reveals that in NAFLD patients without liver fibrosis, eGFR is negatively correlated with NHHR and the risk of developing CKD is higher in the group with the highest NHHR compared to the group with the lowest. The RCS curve model results indicate that the risk of developing CKD in NAFLD patients with without liver fibrosis escalates with NHHR values rise. However, for NAFLD patients with liver fibrosis, the impact of NHHR on the risk of developing CKD has changed. In the NAFLD with liver fibrosis group, although the correlation analysis results were negative, new findings were discovered in the RCS curve model results. The RCS curve model results showed a U-shaped relationship between NHHR and CKD in NAFLD patients with liver fibrosis. These findings indicate that although an elevated NHHR is a risk factor for CKD in NAFLD patients, an excessively low NHHR is not beneficial. For patients with NAFLD, maintaining NHHR within an appropriate range can significantly reduce the risk of developing CKD. It also indicates that there are population-based differences in NHHR among patients with NAFLD, and NHHR has different impacts on different groups of patients with NAFLD.

In NAFLD patients without liver fibrosis, subgroup analysis results show that NHHR is more significantly associated with a higher risk of CKD in female patients, a phenomenon that may be closely related to endocrine factors. Multiple studies have pointed out that normal estrogen secretion before menopause is a key mechanism for women to resist the development of NAFLD. One of the core mechanisms of NAFLD is the excessive accumulation of lipids in the liver and the death of liver cells caused by excessive fat accumulation. The presence of estrogen helps to increase tissue sensitivity to insulin and further promotes the oxidation of free fatty acids in the liver, the secretion of VLDL, and the deposition of fat in subcutaneous tissue, inhibiting the deposition of lipids in the liver and achieving the purpose of preventing the occurrence of NAFLD (27, 52). At the same time, studies have shown that estrogen can slow down the progression of kidney disease by dilating renal blood vessels and inhibiting renal interstitial fibrosis (53–55). The average age of the population included in this study is 49 years old, and some female patients may have entered menopause. The sharp decline in estrogen levels during menopause can lead to the loss of estrogen’s protective effects on the liver and kidneys. In addition, a considerable number of NAFLD patients also suffer from diabetes. In women, the occurrence of diabetes is often related to the increase in male hormone levels and the decrease in estrogen levels (56–58). Studies have shown that testosterone can cause kidney function damage by activating the C-jun or fibrotic signaling pathways (59, 60). Therefore, diabetes may be another reason for the higher risk of CKD in female patients. Thirdly, in terms of sex hormone disorders, we cannot ignore the impact of polycystic ovary syndrome on female patients’ hormone levels. One of the specific manifestations of polycystic ovary syndrome is excessive secretion of androgens (61). Studies have shown that polycystic ovary syndrome is associated with the occurrence of CKD, and common comorbidities of polycystic ovary syndrome include diabetes, obesity, and other metabolic-related diseases (62). Therefore, age, diabetes, and sex hormone disorders caused by polycystic ovary syndrome may be the reasons for the correlation between NHHR and a higher risk of CKD in female NAFLD without fibrosis patients.

Additionally, in the subgroup analysis of NAFLD with fibrosis, it was found that NHHR is more significantly associated with a higher risk of CKD in non-diabetic patients. However, in the subgroup analysis of NAFLD without fibrosis, NHHR is more significantly associated with a higher risk of CKD in diabetic patients. The etiology of CKD is multifactorial, involving both unchangeable factors such as age and genetic susceptibility, as well as modifiable factors such as diabetes, hypertension, and obesity. The overlap of risk factors between CKD and NAFLD has been mentioned above. Furthermore, Chang et al. found that in non-hypertensive and non-diabetic NAFLD patients, NAFLD remains an independent risk factor for increased CKD risk (28). Ryu et al. discovered that the biomarkers gamma-glutamyltransferase, which can be used for the diagnosis of NAFLD, can also serve as independent predictors for assessing the risk of CKD in non-hypertensive and non-diabetic patients (63–65). From the above studies, it can be seen that the impact of NAFLD on CKD is independent of metabolic disorders such as diabetes. However, the mediating role of metabolic factors such as insulin resistance and lipid metabolism disorders in the promotion of CKD by NAFLD cannot be overlooked (66, 67). Therefore, both glucose metabolism disorders and NAFLD are involved in the occurrence of CKD, which is a reasonable explanation for the different impacts of diabetes on different NAFLD patient groups. Moreover, the effects of glucose metabolism disorders and NAFLD on CKD are both interconnected and independent.

Our study possesses several limitations. Firstly, this study is a cross-sectional analysis, focusing on an adult population in the United States, which may have population-specific constraints, especially in countries with different epidemiological characteristics of dyslipidemia, NAFLD, and CKD. Secondly, the assessment of liver fibrosis in this study was not based on precise liver biopsies. Thirdly, although the association between NHHR and CKD has been clearly emphasized in this study, it is not possible to determine whether an increase in NHHR directly leads to deterioration of renal function. Longitudinal studies are needed to confirm this causal relationship and further elucidate the biological mechanisms underlying the observed association. Additionally, missing self-reported data or variables in NHANES, which may introduce potential biases. Lastly, this study did not categorize CKD stages, thus it cannot provide a detailed understanding of the impact of NHHR on different stages of CKD in NAFLD patients. Future research is needed to further explore the relationship between NHHR and different stages of CKD.

## 5 Conclusion

In conclusion, our study found a U-shaped relationship between NHHR and CKD in NAFLD patients with liver fibrosis as well as a linear relationship with CKD in NAFLD patients without liver fibrosis. Our findings underscore the practical utility of NHHR as a biomarker for early risk stratification of CKD in patients with NAFLD. Monitoring NHHR may assist in assessing the risk of CKD in patients with NAFLD.

## Data Availability

The original contributions presented in the study are included in the article/supplementary material, further inquiries can be directed to the corresponding author.

## References

[1] Mantovani A Scorletti E Mosca A Alisi A Byrne CD Targher G. Complications, morbidity and mortality of nonalcoholic fatty liver disease. Metabolism. (2020) 111:154170. doi: 10.1016/j.metabol.2020.154170, PMID: 32006558

[2] Younossi ZM Koenig AB Abdelatif D Fazel Y Henry L Wymer M. Global epidemiology of nonalcoholic fatty liver disease-meta-analytic assessment of prevalence, incidence, and outcomes. Hepatology. (2016) 64:73–84. doi: 10.1002/hep.28431, PMID: 26707365

[3] Friedman SL Neuschwander-Tetri BA Rinella M Sanyal AJ. Mechanisms of NAFLD development and therapeutic strategies. Nat Med. (2018) 24:908–22. doi: 10.1038/s41591-018-0104-9, PMID: 29967350 PMC6553468

[4] Byrne CD Targher G. NAFLD: a multisystem disease. J Hepatol. (2015) 62:S47–64. doi: 10.1016/j.jhep.2014.12.012, PMID: 25920090

[5] Targher G Byrne CD. Non-alcoholic fatty liver disease: an emerging driving force in chronic kidney disease. Nat Rev Nephrol. (2017) 13:297–310. doi: 10.1038/nrneph.2017.16, PMID: 28218263

[6] Stevens PE Levin A. Kidney disease: improving global outcomes chronic kidney disease guideline development work group members. Evaluation and management of chronic kidney disease: synopsis of the kidney disease: improving global outcomes 2012 clinical practice guideline. Ann Intern Med. (2013) 158:825–30. doi: 10.7326/0003-4819-158-11-201306040-00007, PMID: 23732715

[7] Targher G Chonchol MB Byrne CD. CKD and nonalcoholic fatty liver disease. Am J Kidney Dis. (2014) 64:638–52. doi: 10.1053/j.ajkd.2014.05.019, PMID: 25085644

[8] Musso G Gambino R Tabibian JH Ekstedt M Kechagias S Hamaguchi M. Association of non-alcoholic fatty liver disease with chronic kidney disease: a systematic review and meta-analysis. PLoS Med. (2014) 11:e1001680. doi: 10.1371/journal.pmed.1001680, PMID: 25050550 PMC4106719

[9] Noels H Lehrke M Vanholder R Jankowski J. Lipoproteins and fatty acids in chronic kidney disease: molecular and metabolic alterations. Nat Rev Nephrol. (2021) 17:528–42. doi: 10.1038/s41581-021-00423-5, PMID: 33972752

[10] Yang M Geng C-A Liu X Guan M. Lipid disorders in NAFLD and chronic kidney disease. Biomedicines. (2021) 9:1405. doi: 10.3390/biomedicines9101405, PMID: 34680522 PMC8533451

[11] Bowe B Xie Y Xian H Balasubramanian S Al-Aly Z. Low levels of high-density lipoprotein cholesterol increase the risk of incident kidney disease and its progression. Kidney Int. (2016) 89:886–96. doi: 10.1016/j.kint.2015.12.034, PMID: 26924057

[12] Nam KH Chang TI Joo YS Kim J Lee S Lee C. Association between serum high-density lipoprotein cholesterol levels and progression of chronic kidney disease: results from the KNOW-CKD. J Am Heart Assoc. (2019) 8:e011162. doi: 10.1161/JAHA.118.011162, PMID: 30859896 PMC6475054

[13] Melsom T Norvik JV Enoksen IT Stefansson V Rismo R Jenssen T. Association of High-Density Lipoprotein Cholesterol with GFR decline in a general nondiabetic population. Kidney Int Rep. (2021) 6:2084–94. doi: 10.1016/j.ekir.2021.05.007, PMID: 34386657 PMC8343778

[14] Rahman M Yang W Akkina S Alper A Anderson AH Appel LJ. Relation of serum lipids and lipoproteins with progression of CKD: the CRIC study. Clin J Am Soc Nephrol. (2014) 9:1190–8. doi: 10.2215/CJN.09320913, PMID: 24832097 PMC4078958

[15] Hodkinson A Tsimpida D Kontopantelis E Rutter MK Mamas MA Panagioti M. Comparative effectiveness of statins on non-high density lipoprotein cholesterol in people with diabetes and at risk of cardiovascular disease: systematic review and network meta-analysis. BMJ. (2022) 376:e067731. doi: 10.1136/bmj-2021-067731, PMID: 35331984 PMC8943592

[16] Qi X Wang S Huang Q Chen X Qiu L Ouyang K. The association between non-high-density lipoprotein cholesterol to high-density lipoprotein cholesterol ratio (NHHR) and risk of depression among US adults: a cross-sectional NHANES study. J Affect Disord. (2024) 344:451–7. doi: 10.1016/j.jad.2023.10.064, PMID: 37838268

[17] Kim SW Jee JH Kim HJ Jin S-M Suh S Bae JC. Non-HDL-cholesterol/HDL-cholesterol is a better predictor of metabolic syndrome and insulin resistance than apolipoprotein B/apolipoprotein A1. Int J Cardiol. (2013) 168:2678–83. doi: 10.1016/j.ijcard.2013.03.027, PMID: 23545148

[18] Cao Y Li P Zhang Y Qiu M Li J Ma S. Association of systemic immune inflammatory index with all-cause and cause-specific mortality in hypertensive individuals: Results from NHANES. Front Immunol. (2023) 14:1087345. 36817427 10.3389/fimmu.2023.1087345 PMC9932782

[19] Eddowes PJ Sasso M Allison M Tsochatzis E Anstee QM Sheridan D. Accuracy of FibroScan controlled attenuation parameter and liver stiffness measurement in assessing steatosis and fibrosis in patients with nonalcoholic fatty liver disease. Gastroenterology. (2019) 156:1717–30. doi: 10.1053/j.gastro.2019.01.042, PMID: 30689971

[20] Harring M Golabi P Paik JM Shah D Racila A Cable R. Sarcopenia among patients with nonalcoholic fatty liver disease (NAFLD) is associated with advanced fibrosis. Clin Gastroenterol Hepatol. (2023) 21:2876–2888.e5. doi: 10.1016/j.cgh.2023.02.013, PMID: 36848980

[21] Hong H He Y Gong Z Feng J Qu Y. The association between non-high-density lipoprotein cholesterol to high-density lipoprotein cholesterol ratio (NHHR) and kidney stones: a cross-sectional study. Lipids Health Dis. (2024) 23:102. doi: 10.1186/s12944-024-02089-x, PMID: 38615008 PMC11015599

[22] Kidney Disease: Improving Global Outcomes (KDIGO) Glomerular Diseases Work Group. KDIGO. Clinical practice guideline for the Management of Glomerular Diseases. Kidney Int. (2021) 100:S1–S276. doi: 10.1016/j.kint.2021.05.021, PMID: 34556256

[23] Ren Y Cai Z Guo C Zhang Y Xu H Liu L. Associations between Life’s essential 8 and chronic kidney disease. J Am Heart Assoc. (2023) 12:e030564. doi: 10.1161/JAHA.123.030564, PMID: 38063194 PMC10863789

[24] Zhu J Wei Y. Exposure to p-dichlorobenzene and serum α-klotho levels among US participants in their middle and late adulthood. Sci Total Environ. (2023) 858:159768. doi: 10.1016/j.scitotenv.2022.159768, PMID: 36309252

[25] Xing W Gao W Zhao Z Xu X Bu H Su H. Dietary flavonoids intake contributes to delay biological aging process: analysis from NHANES dataset. J Transl Med. (2023) 21:492. doi: 10.1186/s12967-023-04321-1, PMID: 37480074 PMC10362762

[26] Carrero JJ Hecking M Chesnaye NC Jager KJ. Sex and gender disparities in the epidemiology and outcomes of chronic kidney disease. Nat Rev Nephrol. (2018) 14:151–64. doi: 10.1038/nrneph.2017.181, PMID: 29355169

[27] Lonardo A Nascimbeni F Ballestri S Fairweather D Win S Than TA. Sex differences in nonalcoholic fatty liver disease: state of the art and identification of research gaps. Hepatology. (2019) 70:1457–69. doi: 10.1002/hep.30626, PMID: 30924946 PMC6766425

[28] Chang Y Ryu S Sung E Woo H-Y Oh E Cha K. Nonalcoholic fatty liver disease predicts chronic kidney disease in nonhypertensive and nondiabetic Korean men. Metabolism. (2008) 57:569–76. doi: 10.1016/j.metabol.2007.11.022, PMID: 18328362

[29] Mantovani A Petracca G Beatrice G Csermely A Lonardo A Schattenberg JM. Non-alcoholic fatty liver disease and risk of incident chronic kidney disease: an updated meta-analysis. Gut. (2022) 71:156–62. doi: 10.1136/gutjnl-2020-323082, PMID: 33303564

[30] Targher G Bertolini L Rodella S Zoppini G Lippi G Day C. Non-alcoholic fatty liver disease is independently associated with an increased prevalence of chronic kidney disease and proliferative/laser-treated retinopathy in type 2 diabetic patients. Diabetologia. (2008) 51:444–50. doi: 10.1007/s00125-007-0897-4, PMID: 18058083

[31] Bessone F Razori MV Roma MG. Molecular pathways of nonalcoholic fatty liver disease development and progression. Cell Mol Life Sci. (2019) 76:99–128. doi: 10.1007/s00018-018-2947-0, PMID: 30343320 PMC11105781

[32] Masoodi M Gastaldelli A Hyötyläinen T Arretxe E Alonso C Gaggini M. Metabolomics and lipidomics in NAFLD: biomarkers and non-invasive diagnostic tests. Nat Rev Gastroenterol Hepatol. (2021) 18:835–56. doi: 10.1038/s41575-021-00502-9, PMID: 34508238

[33] Heeren J Scheja L. Metabolic-associated fatty liver disease and lipoprotein metabolism. Mol Metab. (2021) 50:101238. doi: 10.1016/j.molmet.2021.101238, PMID: 33892169 PMC8324684

[34] Jiang ZG de Boer IH Mackey RH Jensen MK Lai M Robson SC. Associations of insulin resistance, inflammation and liver synthetic function with very low-density lipoprotein: the cardiovascular health study. Metabolism. (2016) 65:92–9. doi: 10.1016/j.metabol.2015.10.017, PMID: 26892520 PMC4761104

[35] Kawano Y Cohen DE. Mechanisms of hepatic triglyceride accumulation in non-alcoholic fatty liver disease. J Gastroenterol. (2013) 48:434–41. doi: 10.1007/s00535-013-0758-5, PMID: 23397118 PMC3633701

[36] Young SG Zechner R. Biochemistry and pathophysiology of intravascular and intracellular lipolysis. Genes Dev. (2013) 27:459–84. doi: 10.1101/gad.209296.112, PMID: 23475957 PMC3605461

[37] Cohen DE Fisher EA. Lipoprotein metabolism, dyslipidemia, and nonalcoholic fatty liver disease. Semin Liver Dis. (2013) 33:380–8. doi: 10.1055/s-0033-1358519, PMID: 24222095 PMC3988578

[38] Speliotes EK Massaro JM Hoffmann U Vasan RS Meigs JB Sahani DV. Fatty liver is associated with dyslipidemia and dysglycemia independent of visceral fat: the Framingham heart study. Hepatology. (2010) 51:1979–87. doi: 10.1002/hep.23593, PMID: 20336705 PMC3023160

[39] Katsiki N Mikhailidis DP Mantzoros CS. Non-alcoholic fatty liver disease and dyslipidemia: an update. Metabolism. (2016) 65:1109–23. doi: 10.1016/j.metabol.2016.05.003, PMID: 27237577

[40] McCullough A Previs SF Dasarathy J Lee K Osme A Kim C. HDL flux is higher in patients with nonalcoholic fatty liver disease. Am J Physiol Endocrinol Metab. (2019) 317:E852–62. doi: 10.1152/ajpendo.00193.2019, PMID: 31503515 PMC6879863

[41] Mikolasevic I Milic S Turk Wensveen T Grgic I Jakopcic I Stimac D. Nonalcoholic fatty liver disease – a multisystem disease? World J Gastroenterol. (2016) 22:9488–505. doi: 10.3748/wjg.v22.i43.9488, PMID: 27920470 PMC5116593

[42] Park H Dawwas GK Liu X Nguyen MH. Nonalcoholic fatty liver disease increases risk of incident advanced chronic kidney disease: a propensity-matched cohort study. J Intern Med. (2019) 286:711–22. doi: 10.1111/joim.12964, PMID: 31359543 PMC6851415

[43] Byun AR Lee SW Lee HS Shim KW. What is the most appropriate lipid profile ratio predictor for insulin resistance in each sex? A cross-sectional study in Korean populations (the fifth Korea National Health and nutrition examination survey). Diabetol Metab Syndr. (2015) 7:59. doi: 10.1186/s13098-015-0051-2, PMID: 26146523 PMC4491241

[44] Vaziri ND. HDL abnormalities in nephrotic syndrome and chronic kidney disease. Nat Rev Nephrol. (2016) 12:37–47. doi: 10.1038/nrneph.2015.180, PMID: 26568191

[45] Bril F Sninsky JJ Baca AM Superko HR Portillo Sanchez P Biernacki D. Hepatic steatosis and insulin resistance, but not steatohepatitis, promote atherogenic Dyslipidemia in NAFLD. J Clin Endocrinol Metab. (2016) 101:644–52. doi: 10.1210/jc.2015-3111, PMID: 26672634

[46] Deprince A Haas JT Staels B. Dysregulated lipid metabolism links NAFLD to cardiovascular disease. Mol Metab. (2020) 42:101092. doi: 10.1016/j.molmet.2020.101092, PMID: 33010471 PMC7600388

[47] Tani M Kawakami A Mizuno Y Imase R Ito Y Kondo K. Small dense LDL enhances THP-1 macrophage foam cell formation. J Atheroscler Thromb. (2011) 18:698–704. doi: 10.5551/jat.7161, PMID: 21512280

[48] Kamanna VS Pai R Ha H Kirschenbaum MA Roh DD. Oxidized low-density lipoprotein stimulates monocyte adhesion to glomerular endothelial cells. Kidney Int. (1999) 55:2192–202. doi: 10.1046/j.1523-1755.1999.00470.x, PMID: 10354268

[49] Lee HS Song CY. Oxidized low-density lipoprotein and oxidative stress in the development of glomerulosclerosis. Am J Nephrol. (2009) 29:62–70. doi: 10.1159/000151277, PMID: 18689980

[50] Satirapoj B Bruhn KW Nast CC Wang Y Dai T Lapage J. Oxidized low-density lipoprotein antigen transport induces autoimmunity in the renal tubulointerstitium. Am J Nephrol. (2012) 35:520–30. doi: 10.1159/000338484 22653259

[51] Sung P-H Cheng B-C Hsu T-W Chiang JY Chiang H-J Chen Y-L. Oxidized-LDL deteriorated the renal residual function and parenchyma in CKD rat through upregulating epithelial mesenchymal transition and extracellular matrix-mediated tubulointerstitial fibrosis-pharmacomodulation of rosuvastatin. Antioxidants (Basel). (2022) 11:2465. doi: 10.3390/antiox11122465, PMID: 36552673 PMC9774560

[52] Chen H Liu Y Liu D Liang Y Zhu Z Dong K. Sex- and age-specific associations between abdominal fat and non-alcoholic fatty liver disease: a prospective cohort study. J Mol Cell Biol. (2024) 15:mjad069. doi: 10.1093/jmcb/mjad069, PMID: 38037475 PMC11161703

[53] Cao R Su W Sheng J Guo Y Su J Zhang C. Estrogen receptor β attenuates renal fibrosis by suppressing the transcriptional activity of Smad3. Biochim Biophys Acta Mol basis Dis. (2023) 1869:166755. doi: 10.1016/j.bbadis.2023.166755, PMID: 37196860

[54] Dixon A Maric C. 17beta-Estradiol attenuates diabetic kidney disease by regulating extracellular matrix and transforming growth factor-beta protein expression and signaling. Am J Physiol Renal Physiol. (2007) 293:F1678–90. doi: 10.1152/ajprenal.00079.2007, PMID: 17686959 PMC3179625

[55] Maric C Sandberg K Hinojosa-Laborde C. Glomerulosclerosis and tubulointerstitial fibrosis are attenuated with 17beta-estradiol in the aging dahl salt sensitive rat. J Am Soc Nephrol. (2004) 15:1546–56. doi: 10.1097/01.asn.0000128219.65330.ea, PMID: 15153565

[56] Faltas CL LeBron KA Holz MK. Unconventional Estrogen Signaling in health and disease. Endocrinology. (2020) 161:bqaa030. doi: 10.1210/endocr/bqaa030, PMID: 32128594 PMC7101056

[57] Maggio M Lauretani F Ceda GP Bandinelli S Basaria S Paolisso G. Association of hormonal dysregulation with metabolic syndrome in older women: data from the InCHIANTI study. Am J Physiol Endocrinol Metab. (2007) 292:E353–8. doi: 10.1152/ajpendo.00339.2006, PMID: 16968811 PMC2645662

[58] Oh J-Y Barrett-Connor E Wedick NM Wingard DL Rancho Bernardo Study. Endogenous sex hormones and the development of type 2 diabetes in older men and women: the rancho Bernardo study. Diabetes Care. (2002) 25:55–60. doi: 10.2337/diacare.25.1.55, PMID: 11772901

[59] Metcalfe PD Leslie JA Campbell MT Meldrum DR Hile KL Meldrum KK. Testosterone exacerbates obstructive renal injury by stimulating TNF-alpha production and increasing proapoptotic and profibrotic signaling. Am J Physiol Endocrinol Metab. (2008) 294:E435–43. doi: 10.1152/ajpendo.00704.2006, PMID: 18073317

[60] Verzola D Villaggio B Procopio V Gandolfo MT Gianiorio F Famà A. Androgen-mediated apoptosis of kidney tubule cells: role of c-Jun amino terminal kinase. Biochem Biophys Res Commun. (2009) 387:531–6. doi: 10.1016/j.bbrc.2009.07.056, PMID: 19615976

[61] Jayasena CN Franks S. The management of patients with polycystic ovary syndrome. Nat Rev Endocrinol. (2014) 10:624–36. doi: 10.1038/nrendo.2014.102 25022814

[62] Du Y Li F Li S Ding L Liu M. Causal relationship between polycystic ovary syndrome and chronic kidney disease: a mendelian randomization study. Front Endocrinol. (2023) 14:1120119. doi: 10.3389/fendo.2023.1120119, PMID: 37008943 PMC10050750

[63] Pajuelo-Vasquez R Benites-Meza JK Durango-Chavez HV Salinas-Sedo G Toro-Huamanchumo CJ. Diagnostic performance of the GGT/HDL-C ratio for NAFLD in adults with obesity undergoing bariatric surgery. Diabetes Res Clin Pract. (2024) 211:111649. doi: 10.1016/j.diabres.2024.111649, PMID: 38574896

[64] Ryu S Chang Y Kim D-I Kim WS Suh B-S. Gamma-Glutamyltransferase as a predictor of chronic kidney disease in nonhypertensive and nondiabetic Korean men. Clin Chem. (2007) 53:71–7. doi: 10.1373/clinchem.2006.078980, PMID: 17110470

[65] Xie Q Lu S Kuang M He S Yu C Hu C. Assessing the longitudinal association between the GGT/HDL-C ratio and NAFLD: a cohort study in a non-obese Chinese population. BMC Gastroenterol. (2022) 22:500. doi: 10.1186/s12876-022-02598-y, PMID: 36471271 PMC9724423

[66] Byrne CD Targher G. NAFLD as a driver of chronic kidney disease. J Hepatol. (2020) 72:785–801. doi: 10.1016/j.jhep.2020.01.013, PMID: 32059982

[67] Wang T-Y Wang R-F Bu Z-Y Targher G Byrne CD Sun D-Q. Association of metabolic dysfunction-associated fatty liver disease with kidney disease. Nat Rev Nephrol. (2022) 18:259–68. doi: 10.1038/s41581-021-00519-y, PMID: 35013596

